# Comparing Alternative Approaches to Care Management Prioritization: A Prospective Comparative Cohort Study of Acute Care Utilization and Equity Among Medicaid Beneficiaries

**DOI:** 10.1111/1475-6773.70113

**Published:** 2026-04-04

**Authors:** Parth Sheth, Scott Anders, Sanjay Basu, Aaron Baum, Sadiq Y. Patel

**Affiliations:** ^1^ Clinical Product Development, Waymark Care San Francisco California USA; ^2^ School of Engineering and Applied Sciences, University of Pennsylvania Philadelphia Pennsylvania USA; ^3^ School of Social Policy and Practice, University of Pennsylvania Philadelphia Pennsylvania USA; ^4^ Value Based Care, Providence Health and Services Seattle Washington USA; ^5^ San Francisco General Hospital, University of California San Francisco San Francisco California USA; ^6^ Sloan School of Management, MIT Cambridge Massachusetts USA

## Abstract

**Objective:**

To test whether prioritizing Medicaid beneficiaries for outreach by population health teams based on predicted benefit from care management services reduces acute care utilization more effectively than traditional prioritization approaches based on predicted risk of future acute care utilization.

**Data Sources/Study Setting:**

Healthcare utilization data among 9266 adult Medicaid beneficiaries assigned to primary care practices in Washington State (May–December 2024).

**Study Design:**

Prospective cohort study. A difference‐in‐differences analysis was conducted to compare patients between two clusters of clinics using alternative prioritization strategies for population health care management outreach: one cluster using risk‐based prioritization (*N* = 4572 patients prioritized by predicted probability of future acute care utilization without intervention), while the other changed from risk‐based to benefit‐based prioritization (*N* = 4694 prioritized by predicted reduction in probability of future acute care utilization with versus without intervention).

**Data Collection/Extraction Methods:**

Claims data were collected from the Medicaid health plans of patients, while emergency visit and hospitalization notices were obtained from an admit, discharge and transfer data feed service.

**Principal Findings:**

In the intention‐to‐treat analysis of all 9266 patients, benefit‐based prioritization reduced acute care visits by 92.4 per 1000 member‐months compared with risk‐based prioritization (95% CI: −113.0, −72.0; *p* < 0.001). Among the 2845 successfully engaged patients, benefit‐based prioritization was associated with 208.4 fewer visits per 1000 member‐months (95% CI: −284.0, −133.0; *p* < 0.001). Outreach and engagement rates were similar between groups, and effects were also consistent across sex and race/ethnicity subgroups.

**Conclusions:**

Prioritizing Medicaid care management outreach based on predicted benefit from such services, rather than predicted risk of acute care utilization alone, was associated with similar engagement rates but substantially lower acute care utilization. This approach may improve the effectiveness of resource‐constrained Medicaid programs.

## Summary

- What is known on this topic
  ○ Medicaid care management programs are mandated by state agencies to prevent avoidable emergency department visits and hospitalizations, yet effectiveness has varied widely across randomized trials.
  ○ Mixed trial results suggest that variations in patient population selection, rather than just operational differences, may critically affect care management program effectiveness.
  ○ Whether to prioritize patient outreach based on predicted risk of future utilization or predicted benefit from care management services remains an unresolved question with equity implications.
- What this study adds
  ○ In a prospective matched cohort comparison among high‐risk Medicaid beneficiaries, benefit‐based prioritization reduced acute care utilization more effectively than traditional risk‐based prioritization.
  ○ Benefit‐based prioritization identified patients at earlier, modifiable points in their care trajectories, such as after recent medication changes or insurance coverage denials.
  ○ Both prioritization approaches had similar outreach and engagement rates, and neither showed detectable algorithmic bias by sex or race/ethnicity in subgroup analyses.

## 1 Introduction

Medicaid remains the largest healthcare safety net program in the United States, serving over 80 million Americans who are disproportionately of lower income and higher disability than the general population [1]. Nearly all state Medicaid programs require health plans and/or their contracted providers and health systems to offer proactive care management services, which usually involve nurses, community health workers, and other care team members proactively contacting patients who may be experiencing barriers to primary care, inadequate social support, or have uncontrolled chronic conditions. Medicaid care management programs typically target approximately 3%–5% of enrollees with high medical or social complexity—typically by using risk scores that predict future cost or future emergency department or hospital utilization—offering standardized, structured care coordination, medication adherence counseling, and social needs assistance [2, 3, 4]. The common mandate for care management in Medicaid reflects a critical challenge: Medicaid beneficiaries experience the highest rates of preventable emergency department visits, hospitalizations, and costs among insured populations, with approximately 40% of visits deemed “ambulatory‐care sensitive” (potentially addressable through timely and adequate‐quality primary care) [5].

Care management programs typically follow standardized operating protocols and workflows, yet randomized trials of these programs have varied dramatically in their results, with some trials revealing substantial reductions in emergency department visits and hospitalizations [2, 3, 4], while others have shown minimal or null effects [6, 7, 8]. For example, randomized evaluations in settings as different as suburban California, rural Tennessee, and urban Philadelphia have reported large and statistically significant reductions in emergency department visits and hospitalizations [2, 3, 4], while an oft‐cited randomized evaluation of the Camden Coalition's nationally recognized program found no such effect [6], nor did several other randomized studies [7, 9]. A leading untested hypothesis is that the effectiveness of care management depends not only on operational excellence and protocols, but also on patient selection and population heterogeneity [10]. Differences in patient populations, in addition to intervention intensity and duration and setting, likely contribute to variations in effectiveness, begging the question of whether and how to institute more targeted approaches.

A key explanation for mixed findings among randomized trials is heterogeneous treatment effects (HTEs)—systematic variations in how different patients respond to the same set of interventions [11, 12]. A post hoc analysis of one null trial suggested that even when average effects are null, certain highly engaged patient subgroups benefited substantially from care management services [13]. Most care management programs, however, do not proactively identify which patients would benefit most from their specific services, instead relying on risk scores that rank patients by predicted risk of future cost or utilization [14]. While this approach targets those at highest overall risk, it does not consider impactability—whether the patient is actually likely to respond to care management in ways that reduce utilization.

From a clinical perspective, risk‐based prioritization has notable limitations. A critically ill, comatose patient on mechanical ventilation may have high predicted future costs but little capacity to benefit from care management until they are much further in their recovery process. A patient receiving advanced cancer care at a comprehensive center may already have robust coordination in place, making additional outreach from a health plan or third party provider redundant or even confusing. By contrast, a patient newly started on insulin or anticoagulants, or facing insurance barriers at a pharmacy, may be at a critical juncture where targeted support could prevent complications—even if their predicted utilization or costs are more modest.

Advances in the science of detecting and measuring HTEs [15, 16, 17] offer a potential strategy to address unanswered questions in the field of care management prioritization. Machine learning methods now enable more consistent, precise, and transparent identification of patients most likely to benefit from a given intervention by flexibly modeling complex, multidimensional patterns of treatment effect heterogeneity without requiring prespecified interaction terms. While these new methods have been applied to pharmacological studies, the methods have not been previously applied to care management programs widely deployed across Medicaid [18]. Machine learning methods are commonly used for risk prediction, but not yet for benefit prediction in the care management field.

In the current study, we sought to address our gap in knowledge by rigorously comparing risk‐based to benefit‐based care management prioritization in a prospective real‐world implementation study. By comparing these two approaches head‐to‐head among care management teams serving a large Medicaid population, we tested whether prioritizing patients based on predicted benefit—rather than risk alone—can improve the effectiveness and equity of care management programs, offering a pathway to optimize limited resources while addressing persistent disparities in acute care use.

## 2 Methods

This study followed the Strengthening the Reporting of Observational Studies in Epidemiology (STROBE) reporting guideline (see Supplement in Supporting Information for checklist) [19].

### 2.1 Study Setting and Design

We conducted a prospective study comparing two cohorts, to evaluate the two alternative outreach prioritization strategies for Medicaid care management across primary care clinics in the Providence Health System in Seattle, Washington. Care management services were delivered through a partnership between Providence and Waymark, a public benefit organization providing proactive care management services free to patients and their physicians/associated health systems; personnel and technology costs were paid through shared savings arrangements with health plans, in which reductions in emergency room visits and hospitalizations pay for the community health workers, pharmacists, care coordinators, therapists and associated overhead expenditures to operate the program. Care management service delivery was provided as a “wrap around” community‐based service supporting each patient's assigned primary care clinic. The care management program followed a standardized national protocol [20], delivered by a multidisciplinary team of community health workers, social worker therapists, clinical pharmacists, and care coordinators. Services included initial outreach via phone, text, or in‐person contact to establish rapport and assess needs; development of individualized care plans based on patient‐identified goals; regular follow‐up based on patient acuity; pharmacist‐supported medication adherence counseling and prescription‐denial or prior authorization resolution services; care coordination such as appointment scheduling and transportation assistance; cognitive behavioral therapy delivered by licensed therapists; and assistance with social needs applications and referrals such as for food and housing assistance. Many of the care management services were delivered outside of the clinic or hospital setting, in community settings such as barber shops, food banks, and homeless shelters. Details of the protocols and operations were published previously [20].

We performed a difference‐in‐differences analysis to compare one group of patients whose primary care clinics and associated care management services remained on risk‐based prioritization and a second group of patients whose clinics and services converted from risk‐based to benefit‐based prioritization. Difference‐in‐differences analyses compare groups with similar pre‐policy change trends in an outcome (in our case, acute care visits defined as the combination of emergency department visits and hospitalizations) to evaluate the significance of a post‐policy change in one group versus the other, controlling for secular trends and time‐invariant unmeasured confounders (such as unmeasured or unobserved differences between the patient groups or their clinics). All participating clinics in the current study initially used risk‐based prioritization during the pre‐policy change period (May–August 2024), targeting the top 30% of all patients, ranked by predicted probability of acute care visits within 120 days. The top 30% of patients were targeted due to constraints to capacity and were consistent across both arms of the study. This approach established a uniformly high‐risk denominator of patients across all sites for consistent baseline comparison.

In the post‐policy change period (September–December 2024), one group of clinics and their associated care management services shifted from risk‐based to benefit‐based prioritization. Assignment to prioritization strategy was not randomized but was jointly determined by Providence clinics and Waymark leadership in partnership, as part of coordinated service planning and rollout. This was not a cluster randomized trial but a pragmatic implementation design based on operational readiness.

Importantly, both prioritization strategies continued to focus on the same top 30% high‐risk denominator identified during the pre‐period. In the benefit‐based clinics, patients within the 30% high‐risk patient group were re‐ranked based on predicted reductions in acute care use achievable through care management (i.e., individualized benefit from care management services), while the risk‐based clinics continued to rank patients solely by predicted risk of utilization. This design, alongside the difference‐in‐differences statistical method to correct for baseline unmeasured confounders differing between the clinics, was chosen to calculate differences in outcomes reflecting the prioritization strategy rather than differences in the underlying high‐risk populations or their clinics, which were further addressed through matching and balance checks in the analysis (detailed below in the Section 2.7).

This pragmatic approach allowed the health system to evaluate benefit‐based prioritization under real‐world conditions while maintaining ongoing risk‐based prioritization at other clinics. Care management staff remained blinded to the underlying prioritization strategy: each month they received ranked lists of patients for outreach without knowing which algorithm generated them. Standardized outreach protocols and care management services were maintained across all sites, with staff instructed to prioritize outreach to the top 30% of patients on each monthly list to ensure consistent targeting intensity across study arms.

### 2.2 Study Population

Eligible participants were adult Medicaid beneficiaries enrolled from May 1 to December 31, 2024. The study cohort was restricted to the top 30% of patients based on predicted risk of acute care utilization, ensuring both benefit‐based and risk‐based strategies targeted the same high‐risk group to isolate the effect of differences in prioritization among the high‐risk population. Patients who were dually eligible for Medicare, lived in long‐term care facilities, or received hospice care were excluded as such patients were often eligible for other simultaneous care management services.

### 2.3 Data Collection

Real‐time acute care utilization (admit/discharge/transfer [ADT]) data were received from all emergency departments and hospitals statewide. Successful engagement was defined as at least one completed care management encounter documented in the program's electronic health record.

### 2.4 Alternative Prioritization Strategies

#### 2.4.1 Risk‐Based Prioritization

Risk‐based prioritization was performed by using a previously validated, peer‐reviewed, and published open‐source machine learning risk‐prediction score using a standard XGBoost machine learning model (one of the most common risk‐modeling methods) with 92% accuracy for predicting emergency department visits or hospitalizations in the next 6 months in a national Medicaid sample [21]. The score incorporated claims‐based variables including demographics, chronic conditions, prior utilization, prescription medications, medication adherence, and acute care visits. Model code is available at https://github.com/sadiqypatel/Medicaid_Risk_Model. Within the top 30% high‐risk cohort, patients were ranked by predicted risk of acute care utilization to prioritize outreach.

#### 2.4.2 Benefit‐Based Prioritization

Benefit‐based prioritization was performed by developing and validating a generalized random forest algorithm (GRF, specifically “causal forests”) to estimate individual treatment effects (ITEs)—the predicted personalized reduction in acute care visits attributable to successful care management engagement. GRF was selected because it is the current state‐of‐the‐art approach to ITE estimation, as it addresses key challenges in the field: handling high‐dimensional data, modeling complex nonlinear relationships, providing robust uncertainty quantification, and performing as well as or better than alternative methods ranging from simple linear regressions to advanced neural networks in independent comparisons [16, 17, 22, 23, 24].

Unlike conventional subgroup analyses that test prespecified interactions, GRF recursively partitions data to identify combinations of variables that predict heterogeneous responses to treatment. This approach has been shown to detect complex treatment effect patterns in healthcare claims missed by traditional regression methods [16, 17, 22, 23, 24]. Within the same top 30% high‐risk cohort, patients were ranked by predicted benefit to prioritize outreach.

The GRF model was trained on historical data from the pre‐intervention period using the following predictors: demographics (age, sex, race/ethnicity), medication count (unique National Drug Codes [NDC]), medication adherence (mean medication possession ratio [MPR] across NDCs), number of clinical conditions (unique Clinical Classification Software Refined codes), chronic conditions (Elixhauser Comorbidities from ICD‐10 codes), number of primary care visits, number of acute care visits, risk‐based prediction score (as in the risk‐based group), enrollment duration, and month of patient outreach. The model estimated ITEs as the difference between predicted outcomes under care management versus no care management for each patient. Hence, the risk‐prediction score was included as one of several features to allow conditioning on baseline risk while identifying differential responsiveness to care management.

Ground truth labels for model training were based on observed changes in acute care utilization following engagement versus no engagement, using historical data from the pre‐policy change period (May–August 2024). The model was trained on the full population eligible for care management during the pre‐policy change period. Validation was conducted on a heldout subset, distinct from the studied cohort used to assess benefit‐based prioritization impact. The heldout validation set comprised 20% of the pre‐intervention data, stratified to maintain the same distribution of outcomes and key predictors as the training set. Model performance was assessed using area under the receiver operating characteristic curve (AUC) for classification tasks and mean squared error (MSE) for prediction tasks (see Table S1).

After model convergence, discrimination and calibration were assessed using a heldout sample, following standards for HTE detection [7]. Model performance was additionally assessed by comparing predicted versus observed reductions in utilization among engaged patients. Patients were then ranked from highest to lowest predicted benefit for outreach prioritization. Model code is available at https://github.com/sadiqypatel/benefit‐based‐hte‐algorithm. Analyses were conducted in Python (v3.13.1) using the open‐source EconML package, which implements a GRF approach with a doubly robust algorithm to mitigate bias from potential unmeasured confounding [25].

### 2.5 Patient Prioritization and Outreach

**FIGURE 1:**
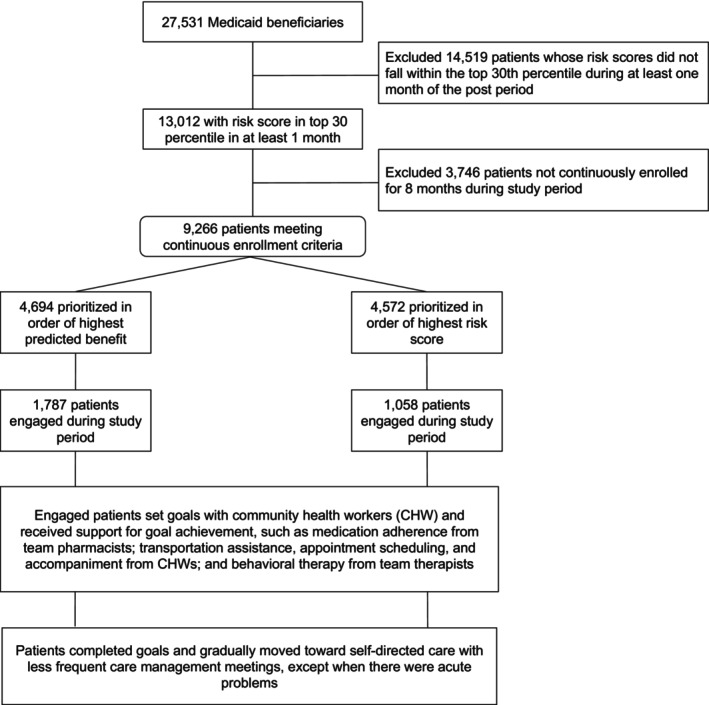
Patient prioritization and outreach diagram. Flow diagram illustrating the care management outreach process, depicting how patients were prioritized using either risk‐based or benefit‐based prioritization, the subsequent outreach methods employed, and resulting patient engagement and service pathways.

The eligible participant pool constituted 27,531 Medicaid beneficiaries, of which 9266 patients met inclusion criteria (Figure 1) and comprised the intention‐to‐treat (ITT) population (Figure 1). Care management teams, blinded to the prioritization method, attempted outreach through standardized channels including telephone, text, and in‐person contact, and successfully engaged 2845 patients (with engagement defined as having completed at least one documented care management encounter).

### 2.6 Outcomes

The primary outcome was acute care visits per 1000 member‐months, defined as combined emergency department visits and hospitalizations using admit/discharge/transfer data. Secondary outcomes were emergency department visits and hospitalizations analyzed separately, and acute care spending per member per month from healthcare claims, using median cost to avoid bias from outliers or delayed claim adjustments (see Supplement). All costs were expressed in 2024 US dollars.

We used combined acute care visits as the primary outcome because care management interventions often shift utilization between emergency and inpatient settings, making isolated metrics potentially misleading. This composite measure has been used in prior Medicaid care management evaluations and randomized trials (2–4, 20) as a standard indicator of potentially preventable, high‐cost utilization. Both emergency department visits and hospitalizations represent failure points in ambulatory care continuity, and our benefit‐based algorithm—like the model validated in NEJM Catalyst (20)—was specifically trained on combined acute care utilization to capture the total burden of preventable acute events. While we report ED visits and hospitalizations separately as secondary outcomes, the composite measure provides the most comprehensive assessment of care management impact.

### 2.7 Matching

Patients were matched across treatment and control groups using full matching [26, 27, 28], creating sets with either one treated and multiple comparison individuals or vice versa [29]. Full matching reduces bias from observed confounders and potentially from unobserved confounders correlated with them [30, 31]. Full matching was chosen over alternative matching methods, such as propensity score or exact matching, for its flexibility in optimizing balance and minimizing bias, particularly in high‐dimensional covariate settings [27]. Patients were matched on age, sex, race/ethnicity, and counterfactual (pre‐study period) chronic conditions, medication count, and utilization. Matching quality was assessed using standardized mean differences, with values ≤ 0.1 considered acceptable.

### 2.8 Statistical Analysis

Our approach combined difference‐in‐differences estimation with matched cohort analysis to address both measured and time‐invariant unmeasured confounding such as unmeasured differences between intervention and control clinics [32]. The unit of analysis was the individual patient. Following current difference‐in‐differences reporting guidelines [32], we conducted both ITT analyses of all eligible patients and an average treatment effect on the treated (ATET) analysis among the engaged patient subset, as well as contextualizing the effect size by reporting it relative to the counterfactual rate of events—defined as the post‐intervention event rate among the control group. Continuous covariates were standardized by dividing by two standard deviations for comparability of model coefficients [33]. Models adjusted for age, sex, race/ethnicity, chronic conditions, primary care visits, and medication count. Models included fixed effects for time and assigned clinic, with robust standard errors clustered at the clinic level to account for intra‐clinic correlation and heteroskedasticity [34]. We used the Huber–White sandwich estimator to adjust standard errors for within‐clinic correlation, given that selection into the two groups occurred at the clinic rather than individual level. Model diagnostics confirmed randomness of residuals (see Supplement in Supporting Information).

### 2.9 Clinical Review of Prioritization Rankings

We conducted prespecified subgroup analyses to evaluate how prioritization strategy changed who was selected for highest‐priority outreach. Among the same top 30% high‐risk cohort (patients in benefit‐based clinics with both risk and benefit scores available), we compared the top one‐third of patients under risk‐ versus benefit‐based prioritization for clinical review (approximately the top 10% overall). This analysis was performed to provide clearer clinical insights about how benefit‐based re‐ranking changed which patients were prioritized for outreach among the same high‐risk population. The review was performed by two board‐certified internal medicine physician supervisors of Medicaid care management programs.

### 2.10 Bias and Sensitivity Analysis

As a sensitivity analysis, we measured the *E*‐value for each estimate, which quantifies the minimum strength of association that an unmeasured confounder would need to have with both the intervention assignment and the outcome to fully explain away the observed treatment effect [35]. The *E*‐value is widely recommended in comparative effectiveness research to provide an intuitive benchmark for assessing the plausibility of residual confounding. Larger *E*‐values indicate that substantial unmeasured confounding would be required to negate the observed effects from the experiment.

We also conducted prespecified sensitivity and falsification analyses in which we tested for sensitivity of the analysis to alternative matching specifications. First, we excluded pre‐period outcomes from the matching process then applied the difference‐in‐differences framework, comparing these results to our main analysis that included pre‐period utilization in matching to assess for regression‐to‐the‐mean bias. Second, we repeated the analysis among beneficiaries in the bottom 70% of predicted risk who were not eligible for outreach. This allowed us to test for residual unmeasured confounding just below the intervention threshold to confirm that any observed effects were specific to the targeted high‐risk population and not artifactual.

Finally, to assess generalizability, we compared our study sample to national Transformed Medicaid Statistical Information System (T‐MSIS) data for both Washington State and the broader United States total Medicaid population as of 2023 [36].

## 3 Results

### 3.1 Study Population

Of the 9266 patients who comprised the ITT population, 4572 remained on risk‐based prioritization and 4694 transitioned to benefit‐based prioritization during the post‐policy change period (Figure 1). The study population was 53.2% female with a mean age of 31.9 years (SD 18.9), including 13.8% African American, 18.0% Hispanic, and 51.6% White patients (Table 1). Before matching, the benefit‐based group tended to be older (mean 33.7 vs. 30.1 years), with higher proportions of Black patients (18.5% vs. 8.9%), slightly higher medication use (mean 0.87 vs. 0.82 unique medications), and similar clinical burden (mean 0.61 vs. 0.62 chronic conditions). Primary care utilization was modestly lower in the benefit‐based group (0.44 vs. 0.49 visits per member), while pre‐period acute care utilization was slightly higher (0.094 vs. 0.089 visits per member).

**TABLE 1:** Counterfactual characteristics of study population before and after matching.

	Before matching			After matching		
	Risk‐based (*n* = 4572)	Benefit‐based (*n* = 4694)	SMD	Risk‐based (*n* = 4572)	Benefit‐based (*n* = 4694)	SMD
Age, mean (SD)^a^	30.1 (19.3)	33.7 (18.3)	0.19	34.2 (19.0)	33.7 (18.3)	0.03
Male, no. (%)^a^	2085 (45.6)	2253 (48.0)	0.49	2213 (48.4)	2253 (48.0)	0.01
Race/ethnicity, no. (%)^a^	—	—	—	—	—	—
White	2579 (56.4)	2201 (46.9)	0.19	2167 (47.4)	2201 (46.9)	0.01
Black	407 (8.9)	868 (18.5)	0.25	809 (17.7)	868 (18.5)	0.02
Hispanic	873 (19.1)	803 (17.1)	0.05	745 (16.3)	803 (17.1)	0.02
Number of chronic conditions, mean per person (SD)	0.62 (1.2)	0.61 (1.2)	0.01	0.64 (1.2)	0.61 (1.2)	0.03
Number of unique medications, mean per person (SD)	0.82 (1.9)	0.87 (1.2)	0.03	0.96 (2.0)	0.87 (1.2)	0.05
Number of PCP visits, mean per person (SD)	0.49 (0.9)	0.44 (0.8)	0.05	0.42 (0.8)	0.44 (0.8)	0.02
Number of emergency department visits and hospitalizations, mean per person (SD)	0.089 (0.45)	0.094 (0.30)	0.02	0.096 (0.60)	0.094 (0.30)	0.01

*Note:* Counterfactual demographic and clinical characteristics of Medicaid beneficiaries assigned to clinics using risk‐based versus benefit‐based prioritization for care management outreach, showing standardized mean differences before and after full matching. All values are presented as number (percentage) or mean (SD). Absolute values ≤ 0.1 indicate adequate balance per the study protocol.

Abbreviations: PCP, primary care physician; SMD, standardized mean difference.

^a^ No missing values for race/ethnicity, sex, or age.

Table 1 shows counterfactual characteristics before and after matching for outreached patients; as shown, after matching, the two groups were balanced across observed covariates. Each patient was assigned to only one group based on their clinic's prioritization strategy at initial eligibility, with no overlap or crossover between groups during the study period. Outreach and engagement rates were similar between cohorts (*p* = 0.56 and *p* = 0.78 for differences in outreach and engagement, respectively; Figure 1; Table S2).

### 3.2 Prioritization Model Performance

While the previously‐published risk‐based prioritization model showed 92% accuracy for predicting absolute acute care visit rates by person in the subsequent 6 months [37], the newly‐derived model used for benefit‐based prioritization (a generalized random forest model) achieved 80% accuracy for predicting reductions in acute care visits in the heldout validation patient sample, showed good calibration across predicted benefit deciles (slope 1.08, intercept −0.002), and had consistent performance across demographic subgroups (Figures S1 and S2; Table 2).

**TABLE 2:** Estimated reduction in acute care visits from baseline to post period between the treatment and control group.^a^ ^,^ ^b^

	All cause acute care visits	ED visits	Hospitalizations	ED visit spend	Hospitalization spend	Acute care spend
Intention‐to‐treat analysis (ITE)	92.40 (72.0, 113.0)	76.7 (57.0, 97.0)	15.6 (12.0, 19.0)	$20,095 (14,934, 25,414)	$102,586 (78,912, 124,944)	$122,681 (93,846, 150,358)
Average treatment effect on treated analysis (ATET)	208.4 (144.0, 272.0)	173.0 (99.0, 247.0)	35.4 (26.0, 45.0)	$45,326 (25,938, 64,714)	$232,790 (170,976, 295,920)	$278,116 (196,914, 360,634)

^a^ Expressed as visits per 1000 member months.

^b^ Coefficient on “post × treatment” interaction.

### 3.3 Clinical Differences in Risk‐ Versus Benefit‐Based Prioritization

Using the same patient pool but different ranking methods produced only 20.4% overlap in the top one‐third of patients prioritized, demonstrating that benefit‐based ranking prioritized different patients from risk‐based prioritization (Table S3). Upon clinical review, benefit‐based targeting was found to prioritize patients with earlier‐stage or newer disease diagnoses, fewer comorbidities, but more insurance barriers (such as denied medications), more medication nonadherence, and new social risk factors (e.g., recent loss of food security); conversely, risk‐based ranking focused on those with higher prior acute care use and a heavier burden of multiple chronic conditions (Table S3).

### 3.4 Primary Outcome

**FIGURE 2:**
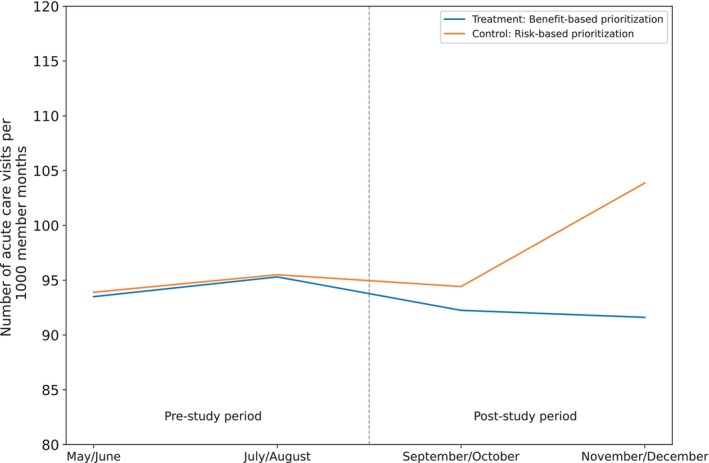
Utilization trends plot. Mean acute care visits per 1000 member‐months in the pre‐ and post‐intervention periods for benefit‐based prioritization (treatment) and risk‐based prioritization (control) patients. During the pre‐study period, trends are parallel and levels are similar between treatment and control patient groups, supporting the parallel trends assumption underlying difference‐in‐differences statistical methods. ATET, averaged treatment effect on the treated; CI, confidence interval; ITT, intention‐to‐treat.

During the pre‐policy change period, the adjusted mean acute care visits per 1000 member‐months were 187.8 in the benefit‐based group and 188.2 in the risk‐based group (*p* = 0.41), demonstrating baseline similarity. Figure 2 visually displays the parallel trends between groups in the pre‐policy change period and the change in the benefit‐based versus risk‐based group in the post‐policy change period.

**FIGURE 3:**
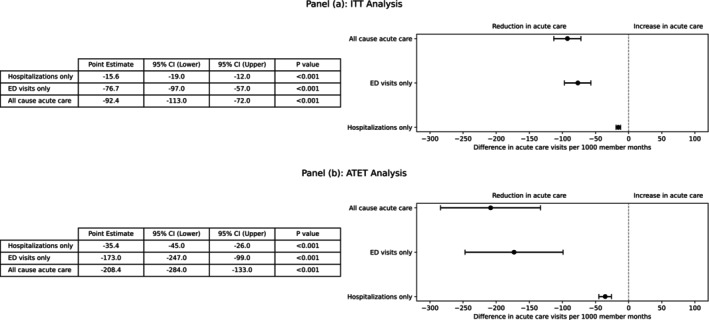
Treatment effects. Forest plot showing difference in acute care visit rates between risk‐based and benefit‐based prioritization cohorts in both intention‐to‐treat and averaged treatment effect on the treated (ATET) analyses. Markers represent point estimates and horizontal lines represent 95% confidence intervals. The vertical dashed line represents no effect. Negative values indicate fewer acute care visits in the benefit‐based prioritization cohort. ATET, averaged treatment effect on the treated; CI, confidence interval; ITT, intention‐to‐treat.

In the ITT analysis (*N* = 9266), the benefit‐prioritized cohort experienced 92.4 fewer acute care visits per 1000 member‐months than the risk‐prioritized cohort in the post‐policy‐intervention (95% CI: −113.0, −72.0; *p* < 0.001), relative to the expected rate of 187.5 visits per 1000 member‐months per the difference‐in‐differences model specification that accounts for secular trends and time‐invariant unmeasured confounders (95% CI: 170.6, 203.6; Figure 3; Table S4).

In the ATET analysis, focused on successfully engaged patients (*N* = 2845), benefit‐based prioritization was associated with 208.4 fewer visits per 1000 member‐months (95% CI: −284.0, −133.0; *p* < 0.001), relative to the expected rate of 356.7 (95% CI: 313.3, 400.0) visits per 1000 member‐months. The ATET estimate (208.4 fewer visits) was more than twice the ITT estimate (92.4 fewer visits), consistent with partial uptake of the intervention (~30% engagement). Both estimates were calculated within the same top 30% high‐risk cohort targeted for outreach in both groups. This limits dilution of the ITT effect and explains its relatively large magnitude despite partial engagement. The large magnitude of both effects reflects the fact that both intervention and comparison groups were composed entirely of high‐risk individuals.

As a falsification test, we repeated the difference‐in‐differences analysis among patients outside the eligible pool (outside the 30% high‐risk cohort targeted for potential outreach) and did not find significant differences in outcomes before or after the policy change period (Table S8).

### 3.5 Secondary Outcomes

In disaggregated outcome analysis, benefit‐based prioritization was associated with reductions in both emergency department visits (−76.8 visits per 1000 member‐months; 95% CI: −97.0, −57.0; *p* < 0.001) relative to the expected value of 159.4, and hospitalizations (−15.6 visits per 1000 member‐months; 95% CI: −19.0, −12.0; *p* < 0.001; Figure 3) relative to the expected value of 28.1 (95% CI: 26.0–30.0). Total medical expenditure was $123 lower per member per month in the benefit‐based group relative to a counterfactual cost of $226 (95% CI: 209, 243; Tables 2, S4, and S5).

### 3.6 Bias and Sensitivity Analysis

**FIGURE 4:**
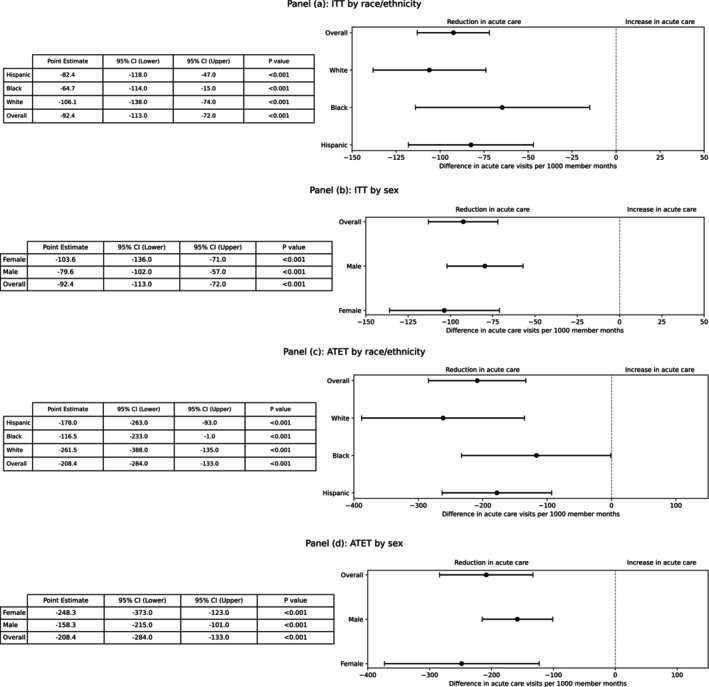
Treatment effects by race/ethnicity and sex. Forest plot showing difference in acute care visit rates between risk‐based and benefit‐based prioritization cohorts in both intention‐to‐treat and averaged treatment effect on the treated (ATET) analyses, by race/ethnic groups and sex. Markers represent point estimates and horizontal lines represent 95% confidence intervals. The vertical dashed line represents no effect. Negative values indicate fewer acute care visits in the benefit‐based prioritization cohort. ATET, averaged treatment effect on the treated; CI, confidence interval; ITT, intention‐to‐treat.

We found no differential effect size estimates by sex or race/ethnicity (Figure 4). Reductions in acute care visits were similar among Black patients (−64.7 visits per 1000 member‐months; 95% CI: −114.0, −15.0), Hispanic patients (−82.4; 95% CI: −118.0, −47.0), and White patients (−106.1; 95% CI: −138.0, −74.0). Equalized‐odds analysis showed no differences in model performance across these subgroups (Supplement Table 7).

The *E*‐value for the primary outcome was 1.5 (lower bound: 1.4) for both ITT and ATET analyses, indicating that an unmeasured confounder would need at least a 1.5‐fold association with both the treatment assignment and the outcome to explain away the results (Table S10). Comparison with national T‐MSIS data showed that the study population was demographically and clinically similar to Washington State and national Medicaid beneficiaries (Table S11). In a falsification analysis among beneficiaries not outreached in either group, there were no significant differences in acute care utilization trends (Table S12). When we reestimated treatment effects using matching methods that excluded pre‐period acute care utilization, results were virtually identical to the main analysis, with an ITT estimate of −92.5 (95% CI: −113.0, −72.0; *p* < 0.001) and ATET estimate of −210.1 (95% CI: −286.0, −134.0; *p* < 0.001; Table S13).

## 4 Discussion

In this prospective comparative cohort study of alternative Medicaid care management prioritization strategies, using predicted benefit rather than predicted risk to prioritize outreach resulted in meaningfully fewer acute care visits, with large effect sizes and narrow confidence intervals in both ITT and engaged‐patient analyses. Outreach and engagement rates were similar between groups, suggesting outcomes were driven by targeting patients more likely to benefit from intervention, not by selecting easier‐to‐reach or easier‐to‐engage patients. Benefit‐based prioritization appeared to identify patients at critical care junctures earlier in their disease journey or with more social or insurance‐related needs—such as those with new diagnoses or medications, insurance denials, or new social service needs—where timely interventions could alter outcomes. In contrast, risk‐based prioritization tended to identify patients with established conditions, multiple comorbidities, often with existing high utilization in hospital settings but fewer social needs. This suggests benefit‐based approaches may help care management programs avoid over‐prioritizing patients whose complexity limits the incremental benefits of a proactive care management team designed to supplement primary care clinics, versus a hospital‐based team for very sick patients, enabling more efficient allocation of scarce resources to those most incrementally likely to benefit from added care management effort. The robustness of our findings was supported by several methodological strengths: the difference‐in‐differences design addressed unmeasured time‐invariant confounding, matching to reduce the risk of measured confounders, sensitivity analyses (*E*‐value) indicated that substantial unmeasured confounding would be required to explain the observed effects, and multiple falsification tests that did not explain away our findings. The benefit‐based approach also had consistent effects across race/ethnic and sex subgroups, which is important when screening for algorithmic bias in machine learning‐driven interventions. The relatively large ITT effect reflects that both groups were drawn from the highest‐risk segment of the Medicaid population. Because intervention and control arms were composed entirely of high‐risk patients, the difference in outcomes was less diluted than in typical ITT analyses. The larger ATET estimate further supports the conclusion that engagement under benefit‐based prioritization yielded substantial reductions in acute care use. The overall cost implications of our results are also substantial, at $123 per person per month in estimated averted costs.

Our findings address a critical challenge in U.S. healthcare delivery. Care management programs, mandated across Medicaid's 80 million patients, represent substantial investments, yet randomized studies show variable effects—from null results [6, 7, 8] to significant reductions in emergency department and hospital utilization [2, 3, 4]. HTEs are a leading explanation [10], and our findings suggest that indeed care management services disproportionately benefited a clinically‐different group than those who may be highest risk for utilization.

This study advances previous research in several ways. t offers a generalizable, open‐source method to identify patients most likely to benefit from a standardized care management program, and demonstrates the feasibility of implementing machine learning‐driven “precision population health” approaches in real‐world settings. While methodological work on HTE modeling has advanced rapidly [15, 16, 17], this is, to our knowledge, the first real‐world application in a proactive population health intervention rather than in pharmacologic studies.

The study has important limitations. It was conducted in a single state and health system, although population characteristics were consistent with national Medicaid data. Because our data sources captured acute care utilization only within Washington State, care received outside the state could not be observed. This unmeasured utilization could introduce bias if out‐of‐state care differed systematically between groups, although such leakage is expected to be minimal given the low likelihood of cross‐state utilization among Medicaid enrollees. While matching and difference‐in‐differences analysis address measured and time‐invariant unmeasured confounding, time‐varying confounding remains possible. This study reflects a pragmatic implementation rather than a randomized design. Clinics were assigned to prioritization strategies based on operational readiness and the availability of sufficient historical outreach data to support benefit‐based modeling. While this enhances external validity, the absence of randomization introduces potential confounding at the clinic level, which may not be fully controlled for among the time‐invariant correction included in the difference‐in‐differences analysis. Additional validation in other states and health systems can help identify how differences in operational capacity or data quality affect the effectiveness of benefit‐based prioritization. Additionally, the algorithm used unique NDC counts as input variables; however, NDCs do not uniquely identify drug products, as identical generic medications in different package sizes have distinct NDCs. Future implementations could use standardized generic drug codes to more accurately capture medication counts.

Several questions merit further research. Qualitative studies could explore why some patients predicted to benefit failed to engage, informing strategies to improve engagement. Future work could also refine matching of specific care management sub‐interventions to patient profiles, further enhancing precision if sufficient statistical power and larger sample sizes become available.

As Medicaid systems face growing resource constraints, optimizing the targeting of services is critical. While risk‐based care management prioritization is the most common approach to date, predicting benefit (i.e., reductions in risk) from available interventions could improve program impact. With rising Medicaid budget pressures, benefit‐based prioritization may offer a way to optimize limited resources.

## Author Contributions

Sadiq Y. Patel had full access to all the data in the study and takes responsibility for the integrity of the data and the accuracy of the data analysis. Design and conduct of the study: Sadiq Y. Patel, Parth Sheth, Sanjay Basu, and Aaron Baum. Acquisition, analysis, or interpretation of data: Sadiq Y. Patel, Parth Sheth, Sanjay Basu, and Aaron Baum. Collection: Sadiq Y. Patel, Parth Sheth, Sanjay Basu, and Aaron Baum. Management: Sadiq Y. Patel. Analysis: Sadiq Y. Patel and Parth Sheth. Interpretation of the data: Sadiq Y. Patel, Parth Sheth, and Sanjay Basu. Preparation: Sadiq Y. Patel, Parth Sheth, and Sanjay Basu. Review: Sadiq Y. Patel, Parth Sheth, Sanjay Basu, Aaron Baum, and Scott Anders. Approval of the manuscript: Sadiq Y. Patel and Sanjay Basu. Decision to submit the manuscript for publication: Sadiq Y. Patel and Sanjay Basu.

## Funding

The authors have nothing to report.

## Conflicts of Interest

All authors except S.A. are employees of Waymark and receive salary and stock options. The algorithms presented here may be of future potential financial benefit to Waymark; hence these authors have potential financial interests in their promotion. S.A. has no conflicts to disclose. S.B. also receives grants from the National Institutes of Health and Centers for Disease Control and Prevention, personal fees from the University of California San Francisco, salary support from HealthRight360, and stock options in Collective Health. Waymark, a public benefit organization, provided all care management services evaluated in this study. Waymark offers care management services to Medicaid beneficiaries at no cost to patients, providers, or health systems; personnel and technology costs are funded through shared savings arrangements with health plans. To support more effective allocation of limited care management resources, Waymark partnered with Providence Health to evaluate the two prioritization approaches compared in this study. No financial incentives were provided to patients or providers based on study participation.

## Supporting information


**Data S1:** hesr70113‐sup‐0001‐Supinfo.docx.

## Data Availability

Research data are not publicly shared due to restrictions from health plans and provider systems.
