# Supplementary material for: Comparing Alternative Approaches to Care Management Prioritization: A Prospective Comparative Cohort Study of Acute Care Utilization and Equity Among Medicaid Beneficiaries
Source: Health Serv Res. 2026 Apr 4;61(2):e70113. doi: 10.1111/1475-6773.70113 (PMC13051954; doi:10.1111/1475-6773.70113)
Supplement: Supplementary file 1 — Data S1: hesr70113‐sup‐0001‐Supinfo.docx. [file HESR-61-e70113-s001.docx]

**Supporting Online Information**

[Supplemental Methods 2](#_gjdgxs)

[Data quality check 2](#_30j0zll)

[Missingness of key variables 2](#_1fob9te)

[Measuring acute care visits 3](#_3znysh7)

[STROBE Reporting Guidelines for Observations Studies 4](#_2et92p0)

[Supplemental Figure 1: Calibration Plot 7](#_3dy6vkm)

[Supplemental Figure 2: Variable Importance Plot 8](#_4d34og8)

[Supplemental Table 1: Model Performance Metrics 10](#_6imjj78stdzl)

[Supplemental Table 2: Outreach and Intervention Rates Comparing Treatment and Control Groups 11](#_xk79d8ne0eyf)

[Supplemental Table 3: Clinical and Demographic Characteristics of Treatment (Benefit-Based, HTE) and Control (Risk-Based, RR) Groups 12](#_z337ya)

[Supplemental Table 4: Percentage change in acute care visits: treatment vs. control group 16](#_1ci93xb)

[Supplemental Table 5: Spending for ED visits and hospitalizations between treatment and control group 18](#_3whwml4)

[Supplemental Table 6: Treatment effect by number of visits 19](#_gtwjixlyq8k8)

[Supplemental Table 7: Performance metrics across demographic groups 20](#_hahe9kg2kezf)

[Supplemental Table 8: Comparison of characteristics among included vs. excluded patients 21](#_bkgondxb32sw)

[Supplemental Table 9: Treatment effect by enrollment criteria 22](#_sws3x1b6jez5)

[Supplemental Table 10: E values: quantifying the impact of unmeasured confounding 23](#_qsh70q)

[Supplemental Table 11: Comparison of study sample to national T-MSIS data 24](#_1pxezwc)

[Supplemental Table 12: Falsification Test 25](#_c5c4vo6prdv0)

[Supplemental Table 13: Sensitivity Analysis: Matching With and Without Pre-Period Acute Care Utilization 25](#_jtjkm1s0xxhq)

## **Supplemental Methods**

### **Data quality check**

We evaluated the completeness of pharmacy and medical claims for the pre-study period (May through August 2024), as baseline predictors were constructed using claims data from this period. Outcomes were measured using ADT data (admit/discharge/transfer feeds) which arrive within 15 minutes via API to the study center. Additionally, we assessed the missingness of key variables required for our analysis.

Completeness of claims data

These values represent the number of claims per member in the Medicaid and pharmacy claims files. We share these figures to highlight the stability and completeness of the claims data used to build the predictors in our modeling.

| Month | Medical claims | Pharmacy claims |
| --- | --- | --- |
| May 2024 | 8.39 | 6.18 |
| June 2024 | 7.66 | 5.13 |
| July 2024 | 8.01 | 5.8 |
| August 2024 | 7.85 | 5.53 |

### **Missingness of key variables**

We applied the DQ Atlas^1^ standard of "low concern," defined as less than 10% missingness for a given variable. Almost all variables had no missing data, except for "primary diagnosis," which was missing for approximately 9.8% of claim lines—still within the DQ Atlas "low concern" threshold.

|  | **Variable** | **% missing** |
| --- | --- | --- |
| **Eligibility** | Medicaid Number | 0% |
|  | Age | 0% |
|  | Sex | 0% |
|  | Enrollment dates | 0% |
| **Pharmacy** | Medicaid Number | 0% |
|  | Claim ID | 0% |
|  | NDC Code | 0% |
|  | Claim status (rejected or paid) | 0% |
|  | Submitted cost | 0% |
|  | Filled date | 0% |
|  | Days supply | 0% |
| **Medical** | Medicaid Number | 0% |
|  | Enrollment dates | 0% |
|  | Procedure codes | 0% |
|  | Primary diagnoses | 9.78% |

### **Measuring acute care visits**

For all-cause acute events, we included emergency department (ED) and inpatient (IP) episodes. Through Point Click Care (PCC, formerly Collective Medical Technologies), we accessed the Washington Health Information Admission/Discharge/Transfer (ADT) data feed for the population included in our study. At the time of this analysis, PCC reported that over 95% of ED and IP visit volume in Washington was covered by their network.

When a patient experienced an ED or IP ADT event in Washington, information about the visit was sent in real time (within 15 minutes) via an HL7 data feed (API). This feed included details such as the patient identifier, event date, event type (admission or emergency visit), an episode grouper identifier (indicating whether two ADT events were related to the same episode), and all associated diagnosis codes.

These data allowed us to prospectively track acute care utilization from a patient’s first to last eligibility within the study and to retrospectively analyze 12 months of acute care utilization data for each patient prior to their eligibility.

## **STROBE Reporting Guidelines for Observations Studies**

|  | Item No | Recommendation | Section |
| --- | --- | --- | --- |
| Title and abstract | 1 | (*a*) Indicate the study’s design with a commonly used term in the title or the abstract | Yes [see title] |
|  |  | (*b*) Provide in the abstract an informative and balanced summary of what was done and what was found | Yes [see abstract] |
| Introduction | | |  |
| Background/  rationale | 2 | Explain the scientific background and rationale for the investigation being reported | Yes [see introduction] |
| Objectives | 3 | State specific objectives, including any prespecified hypotheses | Yes [see introduction] |
| Methods | | |  |
| Study design | 4 | Present key elements of study design early in the paper | Yes [see Methods, Study design and setting] |
| Setting | 5 | Describe the setting, locations, and relevant dates, including periods of recruitment, exposure, follow-up, and data collection | Yes [see Methods, Study design and setting] |
| Participants | 6 | (*a*) Give the eligibility criteria, and the sources and methods of selection of participants | Yes [see Methods, study population] |
| Variables | 7 | Clearly define all outcomes, exposures, predictors, potential confounders, and effect modifiers. Give diagnostic criteria, if applicable | Yes [see Methods, Outcomes] |
| Data sources/ measurement | 8* | For each variable of interest, give sources of data and details of methods of assessment (measurement). Describe comparability of assessment methods if there is more than one group | Yes [see Methods, data collection] |
| Bias | 9 | Describe any efforts to address potential sources of bias | Yes [see Methods, bias and sensitivity analysis] |
| Study size | 10 | Explain how the study size was arrived at | Yes [see Figure 1] |
| Quantitative variables | 11 | Explain how quantitative variables were handled in the analyses. If applicable, describe which groupings were chosen and why | Yes [see Methods, alternative prioritization strategies] |
| Statistical methods | 12 | (*a*) Describe all statistical methods, including those used to control for confounding | Yes [see Methods, Matching and Statistical analysis] |
|  |  | (*b*) Describe any methods used to examine subgroups and interactions | Yes [see Methods, Bias and sensitivity analysis] |
|  |  | (*c*) Explain how missing data were addressed | Yes [see Supplemental Methods section] |
|  |  | (*d*) If applicable, describe analytical methods taking account of sampling strategy | N/A |
|  |  | (*e*) Describe any sensitivity analyses | Yes [see Methods, Bias and sensitivity analysis] |
| Results | | |  |
| Participants | 13* | (a) Report numbers of individuals at each stage of study—eg numbers potentially eligible, examined for eligibility, confirmed eligible, included in the study, completing follow-up, and analysed | Yes [see Results] |
|  |  | (b) Give reasons for non-participation at each stage | Yes [see Figure 1] |
|  |  | (c) Consider use of a flow diagram | Yes [see Figure 1] |
| Descriptive data | 14* | (a) Give characteristics of study participants (eg demographic, clinical, social) and information on exposures and potential confounders | Yes [see Table 1] |
|  |  | (b) Indicate number of participants with missing data for each variable of interest | Yes [see Supplemental Methods] |
| Outcome data | 15* | Report numbers of outcome events or summary measures | Yes [see Results, Figure 2, and Table 2] |
| Main results | 16 | (*a*) Give unadjusted estimates and, if applicable, confounder-adjusted estimates and their precision (eg, 95% confidence interval). Make clear which confounders were adjusted for and why they were included | Yes [see Results, Figure 2, and Table 2] |
|  |  | (*b*) Report category boundaries when continuous variables were categorized | N/A |
|  |  | (*c*) If relevant, consider translating estimates of relative risk into absolute risk for a meaningful time period | N/A |
| Other analyses | 17 | Report other analyses done—eg analyses of subgroups and interactions, and sensitivity analyses | Yes [see Results and Figure 3] |
| Discussion | | |  |
| Key results | 18 | Summarize key results with reference to study objectives | Yes [See Discussion] |
| Limitations | 19 | Discuss limitations of the study, taking into account sources of potential bias or imprecision. Discuss both direction and magnitude of any potential bias | Yes [See Discussion] |
| Interpretation | 20 | Give a cautious overall interpretation of results considering objectives, limitations, multiplicity of analyses, results from similar studies, and other relevant evidence | Yes [See Discussion] |
| Generalisability | 21 | Discuss the generalisability (external validity) of the study results | Yes [See Discussion] |
| Other information | | |  |
| Funding | 22 | Give the source of funding and the role of the funders for the present study and, if applicable, for the original study on which the present article is based | Yes [See Funding] |

##

## **Supplemental Figure 1: Calibration Plot**

Predicted versus observed probability of benefit from care management intervention in deciles of predicted benefit.

##
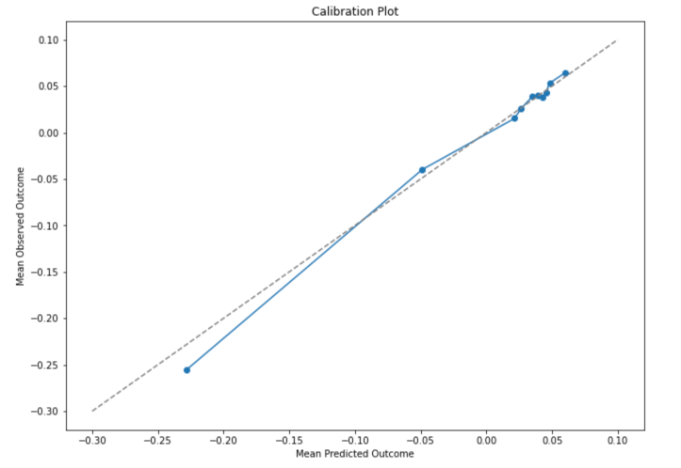


## **Supplemental Figure 2: Variable Importance Plot**

Relative importance of predictor variables in the generalized random forest model. Stage 1 represents propensity model features predicting outreach likelihood, and Stage 2 represents regression model features predicting changes in acute care visits.

The x-axis of SHAP beeswarm plots represents SHAP values, quantifying a feature's impact on model predictions. In stage 1, positive SHAP values indicate an increased predicted likelihood for a patient to be outreached, while in stage 2, they correspond to a predicted increase in acute care visits (negative values indicate a decrease). Dot colors represent feature values (e.g., blue for lower values for a given feature, red for higher values for a given feature), highlighting how variations in feature values influence the predictions.

**Stage 1: Propensity model**

##
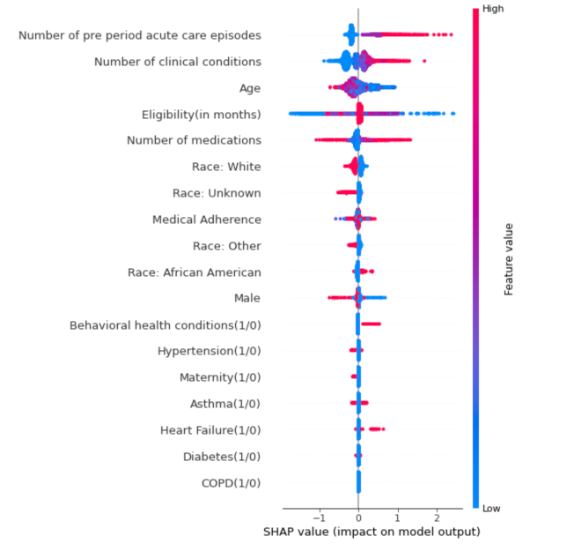


**Stage 2: Regression model**

##
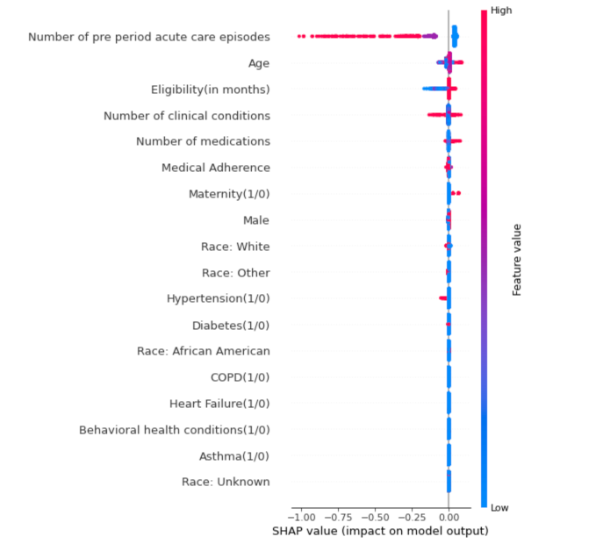


##

##

##

##

## **Supplemental Table 1: Model Performance Metrics**

Detailed metrics of model discrimination, calibration, and stability across subgroups.

| Overall model (ITE prediction) | |
| --- | --- |
| Concordance statistic | 0.71 |
| Stage 1 | |
| Accuracy | 0.81 |
| AUC | 0.58 |
| Stage 2 | |
| MSE | 0.014 |
| R-squared | 0.44 |
| Concordance statistic | 0.73 |
| Calibration | |
| Slope | 1.089 |
| Intercept | -0.002 |
| Plot | see Supplemental Figure 2 |

##

## **Supplemental Table 2: Outreach and Intervention Rates Comparing Treatment and Control Groups**

Outreach is defined as any attempt to contact a given patient, including both successful and unsuccessful attempts. Intervention is defined as delivering care to a patient.

|  | **Outreach** | | **Intervention** | |
| --- | --- | --- | --- | --- |
|  | **Treatment** | **Control** | **Treatment** | **Control** |
|  | Monthly mean of total outreach attempts per week | | Monthly mean of total interventions per week | |
| May 2024 | 200 | 233 | 81 | 97 |
| Jun 2024 | 230 | 240 | 75 | 92 |
| Jul 2024 | 182 | 154 | 65 | 60 |
| Aug 2024 | 145 | 83 | 45 | 34 |
|  | Median number of total outreach attempts per week | | Median number of total interventions per week | |
| Sep 2024 | 219 | 208 | 82 | 104 |
| Oct 2024 | 225 | 237 | 79 | 89 |
| Nov 2024 | 166 | 135 | 61 | 51 |
| Dec 2024 | 124 | 96 | 43 | 37 |

Differences were evaluated overall and separately for the pre- and post-periods

|  | **Outreach** | | | **Intervention** | | |
| --- | --- | --- | --- | --- | --- | --- |
|  | **Overall** | **Pre** | **Post** | **Overall** | **Pre** | **Post** |
| **T-statistic** | 0.58 | -0.58 | 1.75 | -0.28 | -1.34 | 0.85 |
| **P-value** | 0.56 | 0.57 | 0.10 | 0.78 | 0.20 | 0.40 |

##

## **Supplemental Table 3: Clinical and Demographic Characteristics of Treatment (Benefit-Based, HTE) and Control (Risk-Based, RR) Groups**

The goal of this analysis is to assess the percentage overlap in the top 10th percentile of patients—within the top 30th percentile of the total population (i.e., one-third of all patients)—between the Treatment and Control groups. While the overall sample is the same across both groups, we hypothesize that the highest-priority patients will differ due to the distinct prioritization strategies: the benefit-based model selects patients most likely to benefit from our intervention protocols (through reduced acute care visits), whereas the risk-based model prioritizes those at highest risk of acute care utilization, regardless of whether they are likely to benefit from the intervention.

This analysis serves two purposes:

1. **Overlap Analysis**: We evaluated the percentage overlap between risk-based and benefit-based prioritization among the top 10 percentile of patients. Our hypothesis was that the overlap will be smaller, as benefit-based prioritization identifies patients most likely to benefit, as opposed to simply those who are high risk.
2. **Group Characteristics Comparison**: We compared the clinical and demographic characteristics of both groups to highlight how different prioritization strategies lead to distinct group profiles.

**Analysis 1:**

There was a 20.4% overlap.

**Analysis 2:**

|  | **HTE (n = 1248)** | **RR (n = 1169)** |
| --- | --- | --- |
| Mean number of acute care visits | 0.39 | 2.10 |
| Mean number of ED visits | 0.31 | 1.82 |
| Mean number of hospitalizations | 0.08 | 0.28 |
| Mean number of unique medications | 1.10 | 1.80 |
| Mean number of unique clinical conditions | 3.70 | 10.50 |
| Percentage of patients with at least 1 PCP visit | 38.0% | 56.7% |
| Percentage of acute care visits for avoidable conditions | 31.3% | 30.4% |
| Mean medication adherence (proportion days covered) | 95.0% | 91.0% |
| Percentage of patients with increasing slope of acute care visits | 66.0% | 60.0% |

##

| Comorbidity | HTE (n = 1248) | | RR (n = 1169) | | percentage point difference |
| --- | --- | --- | --- | --- | --- |
|  | count | percentage | count | percentage |  |
| At least 1 chronic condition | 321 | 25.72% | 569 | 48.67% | 22.95% |
| Cancer | 22 | 1.76% | 45 | 3.85% | 2.09% |
| Cerebrovascular Disease | 9 | 0.72% | 47 | 4.02% | 3.30% |
| Diabetes, complicated | 54 | 4.33% | 102 | 8.73% | 4.40% |
| Diabetes, uncomplicated | 65 | 5.21% | 141 | 12.06% | 6.85% |
| Hypertension, complicated | 9 | 0.72% | 45 | 3.85% | 3.13% |
| Hypertension, uncomplicated | 86 | 6.89% | 210 | 17.96% | 11.07% |
| Liver failure | 39 | 3.13% | 68 | 5.82% | 2.69% |
| Neurological conditions | 41 | 3.29% | 101 | 8.64% | 5.35% |
| Renal failure | 5 | 0.40% | 27 | 2.31% | 1.91% |
| Thyroid | 44 | 3.53% | 64 | 5.47% | 1.95% |
| Alcohol | 0 | 0.00% | 1 | 0.09% | 0.09% |
| Deficiency anemias | 26 | 2.08% | 85 | 7.27% | 5.19% |
| Auto immune | 12 | 0.96% | 21 | 1.80% | 0.83% |
| Blood loss | 5 | 0.40% | 7 | 0.60% | 0.20% |
| Coagulopathy | 10 | 0.80% | 27 | 2.31% | 1.51% |
| Dementia | 1 | 0.08% | 2 | 0.17% | 0.09% |
| Depression | 4 | 0.32% | 16 | 1.37% | 1.05% |
| Drug abuse | 1 | 0.08% | 2 | 0.17% | 0.09% |
| Heart failure | 10 | 0.80% | 43 | 3.68% | 2.88% |
| Lung chronic | 51 | 4.09% | 103 | 8.81% | 4.72% |
| Obesity | 33 | 2.64% | 73 | 6.24% | 3.60% |
| Paralysis | 10 | 0.80% | 19 | 1.63% | 0.82% |
| Peripheral vascular disease | 8 | 0.64% | 26 | 2.22% | 1.58% |
| Psychoses | 0 | 0.00% | 0 | 0.00% | 0.00% |
| Pulmonary circulation disease | 5 | 0.40% | 13 | 1.11% | 0.71% |
| Peptic ulcer with bleeding | 2 | 0.16% | 4 | 0.34% | 0.18% |
| Valvular disease | 11 | 0.88% | 21 | 1.80% | 0.91% |
| Weight loss | 6 | 0.48% | 25 | 2.14% | 1.66% |

##

Social and clinical goals, as identified for a given patient.

##

| **Goal** | **RR (n = 208)** | **HTE (n = 157)** | **percentage point difference** |
| --- | --- | --- | --- |
| INSURANCE_COVERAGE | 6.40% | 12.04% | -5.63% |
| EMPLOYMENT | 0.49% | 3.70% | -3.21% |
| FINANCIAL | 9.36% | 12.04% | -2.68% |
| DEPRESSION | 0.00% | 1.85% | -1.85% |
| CARE_FOR_MH_BH | 1.48% | 2.78% | -1.30% |
| MEDICATION_OPTIMIZATION | 3.45% | 4.63% | -1.18% |
| ALCOHOL_USE | 0.00% | 0.93% | -0.93% |
| WEIGHT_MANAGEMENT | 0.99% | 1.85% | -0.87% |
| ACTIVITY | 0.49% | 0.93% | -0.43% |
| SOCIAL_CONNECTION | 2.46% | 2.78% | -0.31% |
| PCP_APPOINTMENT | 11.82% | 12.04% | -0.21% |
| POSTPARTUM_CARE | 0.99% | 0.93% | 0.06% |
| EYE_CARE | 2.96% | 2.78% | 0.18% |
| FOOD_INSECURITY | 3.94% | 3.70% | 0.24% |
| LEGAL | 0.49% | 0.00% | 0.49% |
| EDUCATION | 0.49% | 0.00% | 0.49% |
| TECHNOLOGY | 0.49% | 0.00% | 0.49% |
| ASTHMA_COPD | 0.49% | 0.00% | 0.49% |
| CHILDCARE | 0.49% | 0.00% | 0.49% |
| HYPERTENSION | 0.49% | 0.00% | 0.49% |
| DIABETES | 3.45% | 2.78% | 0.67% |
| HEART_FAILURE | 0.99% | 0.00% | 0.99% |
| HOUSING_INSECURITY | 4.93% | 3.70% | 1.22% |
| TRANSPORTATION | 5.42% | 3.70% | 1.72% |
| DENTAL | 7.39% | 5.56% | 1.83% |
| CARE | 11.33% | 9.26% | 2.07% |
| MEDICATION_ADHERENCE | 9.85% | 7.41% | 2.44% |
| OTHER_MENTAL_BEHAVIORAL | 7.39% | 1.85% | 5.54% |

##

## **Supplemental Table 4: Percentage change in acute care visits: treatment vs. control group**

*Measured as visits per 1,000 member-months; spending was measured in 2024 USD and expressed per 1,000 member months.

**Intent-to-treat (ITT) Analysis**

|  | **All cause acute care visits** | **ED visits** | **Hospitalizations** | **ED visit spend** | **Hospitalization spend** | **Acute care spend** |
| --- | --- | --- | --- | --- | --- | --- |
| Baseline mean (visits per 1,000 member months) | 97.6 | 83.6 | 14.0 | $21,914 | $91,989 | $113,904 |
| Expected increase in acute care visits from baseline to post period (coefficient on 'post' main effect) | 89.9 (73.0, 116.0) | 75.8 (60.0, 92.0) | 14.1 (12.0, 16.0) | $19,860 (15720, 24104) | $92,722 (78912, 105216) | $112,581 (94632, 129320) |
| Expected increase in acute care visits during the post period (had there been no intervention) | 187.5 (170.6, 203.6) | 159.4 (143.6, 175.6) | 28.1 (26.0, 30.0) | $41,774 (37623, 46007) | $184,711 (170976, 197280) | $226,485 (208599, 243287) |
| Estimated reduction in acute care visits from baseline to post period (between treatment and control group - coefficient on 'post x treatment' interaction) | 92.40 (72.0, 113.0) | 76.8 (57.0, 97.0) | 15.6 (12.0, 19.0) | $20,095 (14934, 25414) | $102,586 (78912, 124944) | $122,681 (93846, 150358) |

##

**Average Treatment Effect on Treated (ATET) Analysis**

|  | **All cause acute care visits** | **ED visits** | **Hospitalizations** | **ED visit spend** | **Hospitalization spend** | **Acute care spend** |
| --- | --- | --- | --- | --- | --- | --- |
| Baseline mean (visits per 1,000 member months) | 156.3 | 132.9 | 23.4 | $33,667 | $149,275 | $182,942 |
| Expected increase in acute care visits from baseline to post period (coefficient on 'post' main effect) | 200.4 (157.0, 244.0) | 168.7 (100.0, 238.0) | 31.8 (24.0, 39.0) | $44,199 (26200, 62356) | $209,117 (157824, 256464) | $253,316 (184024, 318820) |
| Expected increase in acute care visits during the post period (had there been no intervention) | 356.7 (313.3, 400.3) | 301.6 (232.9, 370.9) | 55.2 (47.4, 62.4) | $77,866 (610202, 97176) | $358,392 (311702, 410,342) | $436,258 (372722, 507518) |
| Estimated reduction in acute care visits from baseline to post period (between treatment and control group - coefficient on 'post x treatment' interaction) | 208.4 (144.0, 272.0) | 173.0 (99.0, 247.0) | 35.4 (26.0, 45.0) | $45,326 (25938, 64714) | $232,790 (170976, 295920) | $278,116 (196914, 360,634) |

##

## **Supplemental Table 5: Spending for ED visits and hospitalizations between treatment and control group**

Our analysis used median spending per episode (in 2024 USD) for ED visits and hospitalizations to evaluate the expected reduction in spending for acute care visits attributable to the benefit-based model compared to the risk-based model. Median spending was chosen due to the high variability in acute care costs. Additionally, we used the median spending across all study sample patients, as it remained consistent across subgroups. For reference, mean spending per episode is included in the table below. Spending is expressed per 1,000 member months.

|  | Mean spend | Median spend |
| --- | --- | --- |
| **Hospitalizations** | | |
| All study sample patients (n = 9266) | **15388** | **6576** |
| Treatment patients | 13972 | 6659 |
| Control patients | 16285 | 6587 |
| **ED visits** | | |
| All study sample patients (n = 9266) | **312** | **262** |
| Treatment patients | 338 | 273 |
| Control patients | 331 | 278 |

##

| Parameter | ED only | | IP only | | All cause acute care | |
| --- | --- | --- | --- | --- | --- | --- |
|  | mean visits | spend | mean visits | spend | mean visits | spend |
| Pre period mean | 80.90 | $21,196 | 13.53 | $88,960 | 94.43 | $110,156 |
| Expected increase from pre to post | 75.80 | $19,860 | 14.10 | $92,722 | 89.90 | $112,581 |
| Expected value of post period mean | 156.70 | $41,056 | 27.63 | $181,681 | 184.33 | $222,737 |
| Expected reduction | 76.70 | $20,095 | 15.60 | $102,586 | 92.40 | $122,681 |

## **Supplemental Table 6: Treatment effect by number of visits**

We split visits into two groups, defined as top 33 percentile and bottom 66 percentile.

##

| **Subgroup identifier** | **Treatment effect** | **Lower CI** | **Upper CI** | **p-value** | **Total N** | **Treatment N** | **Control N** |
| --- | --- | --- | --- | --- | --- | --- | --- |
| Top 33 percentile | -343.2 | -559.0 | -127.0 | 0.002 | 994 | 548 | 446 |
| Bottom 66 percentile | -138.1 | -167.0 | -109.0 | <0.001 | 1706 | 951 | 755 |

##

##

##

## **Supplemental Table 7: Performance metrics across demographic groups**

| **Stability across demographic groups** | | Stage 1 | | Stage 2 | | |
| --- | --- | --- | --- | --- | --- | --- |
|  |  | **Accuracy** | **AUC** | **MSE** | **R-squared** | **Concordance statistic** |
| Gender | Male | 0.82 | 0.57 | 0.014 | 0.44 | 0.72 |
|  | Female | 0.81 | 0.57 | 0.014 | 0.43 | 0.73 |
| Race | White | 0.82 | 0.56 | 0.015 | 0.40 | 0.73 |
|  | Black | 0.79 | 0.60 | 0.017 | 0.53 | 0.71 |
|  | Hispanic | 0.78 | 0.58 | 0.012 | 0.48 | 0.69 |

##

| **Subgroups** | **T value** | **P value** | **Significance** |
| --- | --- | --- | --- |
| White vs Black | -1.38 | 0.169 | No Significant difference |
| White vs Hispanic | -0.97 | 0.331 | No Significant difference |
| Black vs Hispanic | 0.57 | 0.569 | No Significant difference |
|  | | | |
| Male vs Female | 1.19 | 0.234 | No Significant difference |
|  | | | |
| **ATET Comparison** |  |  |  |
| **Subgroups** | **T value** | **P value** | **Significance** |
| White vs Black | -1.66 | 0.098 | No Significant difference |
| White vs Hispanic | -1.07 | 0.283 | No Significant difference |
| Black vs Hispanic | 0.84 | 0.402 | No Significant difference |
|  | | | |
| Male vs Female | 1.28 | 0.199 | No Significant difference |

##

## **Supplemental Table 8: Comparison of characteristics among included vs. excluded patients**

##

|  |  | **With Eligibility Criteria** | | **Without Eligibility Criteria** | |
| --- | --- | --- | --- | --- | --- |
|  |  | **Treatment Group** | **Control Group** | **Treatment Group** | **Control Group** |
| **Size** | Number of patients | 4694 | 4572 | 6275 | 6737 |
| **Age** | mean | 34.54 | 30.89 | 34.05 | 30.24 |
|  | median | 35 | 32 | 35 | 32 |
|  | 25th percentile | 22 | 13 | 22 | 12 |
|  | 75th percentile | 50 | 46 | 49 | 45 |
| **Gender** | percentage female | 52.00% | 54.40% | 52.60% | 55.30% |
|  | percentage male | 48.00% | 45.60% | 47.40% | 44.70% |
| **Race** | percentage Caucasian | 46.90% | 56.40% | 45.40% | 54.80% |
|  | percentage Hispanic | 18.50% | 19.10% | 19.30% | 20.40% |
|  | percentage African American | 17.10% | 8.90% | 17.30% | 8.70% |
|  | percentage Asian | 6.30% | 4.00% | 6.10% | 5.10% |
|  | percentage Unknown | 4.00% | 3.90% | 4.60% | 3.70% |
|  | percentage Other | 2.80% | 2.90% | 2.90% | 2.70% |
|  | percentage Pacific Islander | 2.70% | 2.80% | 2.80% | 2.70% |
|  | percentage Native American | 1.50% | 1.90% | 1.50% | 1.80% |
|  | percentage Native Hawaiian | 0.10% | 0.10% | 0.10% | 0.10% |

##

## **Supplemental Table 9: Treatment effect by enrollment criteria**

| **ITT Analysis** | | | | | | | |
| --- | --- | --- | --- | --- | --- | --- | --- |
| **Eligibility cutoff** | **Treatment effect** | **Lower CI** | **Upper CI** | **p-value** | **Total N** | **Treatment N** | **Control N** |
| >= 1 months (pre and post) | -79.9 | -107.0 | -53.0 | <0.001 | 11927 | 5716 | 6211 |
| >= 2 months (pre and post) | -90.6 | -108.0 | -73.0 | <0.001 | 11186 | 5434 | 5752 |
| >=4 months (pre and post) | -92.4 | -113.0 | -72.0 | <0.001 | 9266 | 4694 | 4572 |
| **ATET Analysis** | | | | | | | |
| **Eligibility cutoff** | **Treatment effect** | **Lower CI** | **Upper CI** | **p-value** | **Total N** | **Treatment N** | **Control N** |
| >= 1 months (pre and post) | -188.3 | -258.0 | -119.0 | <0.001 | 3479 | 2083 | 1396 |
| >= 2 months (pre and post) | -202.6 | -265.0 | -140.0 | <0.001 | 3309 | 2001 | 1308 |
| >=4 months (pre and post) | -208.4 | -272.0 | -144.0 | <0.001 | 2845 | 1787 | 1058 |

## **Supplemental Table 10: E values: quantifying the impact of unmeasured confounding**

*E-value and 95% confidence interval calculated for the treatment × post-period interaction

##

|  | **E-value (95% CI)** |
| --- | --- |
| ITT | 1.49 (1.42-1.55) |
| ATET | 1.50 (1.39-1.61) |

## **Supplemental Table 11: Comparison of study sample to national T-MSIS data**

We used 2019 data to identify rising risk patients.

Abbreviations: SD = standard deviation

|  | **National** | **Washington** |
| --- | --- | --- |
| Age, mean (SD) | 28.9 (18.5) | 29.8 (18.1) |
| Male, No. (%) | 42.4% | 43.7% |
| Race/Ethnicity, No. (%) | – | – |
| White | 42.7% | 52.2% |
| Black | 23.1% | 9.0% |
| Hispanic | 8.6% | 20.5% |
| Number of chronic conditions, mean (SD) | 0.83 (1.3) | 0.92 (1.4) |
| Number of unique medications, mean (SD) | 1.0 (1.5) | 1.3 (1.7) |
| Number of PCP visits, mean (SD) | 0.71 (1.1) | 0.67 (0.9) |
| Number of emergency department visits and hospitalizations, mean (SD) | 1.22 (0.46) | 1.08 (0.39) |

## **Supplemental Table 12: Falsification Test**

As a falsification test, we repeated the analysis among beneficiaries in the bottom 70th percentile of predicted risk (0th–70th percentile) who did not receive outreach, to assess for residual unmeasured confounding. We then further broke down the analysis into two subgroups: the 40th–70th percentile and the 0th–40th percentile of risk. Since our care model targeted only the top 30th percentile, we hypothesized that outcomes in these lower-risk groups would show no effect.

| Risk percentile | Beta | 95% CI | p-value |
| --- | --- | --- | --- |
| 70-100th percentile | -92.4 | [-113.0, -72.0] | <0.001 |
| 0-70th percentile | -10.8 | [-22.0, 0.2] | 0.06 |
| 40-70th percentile | -3.5 | [-7.0, 0.0] | 0.05 |
| 0-40th percentile | -9.2 | [-22.0, 3.0] | 0.17 |

## **Supplemental Table 13: Sensitivity Analysis: Matching With and Without Pre-Period Acute Care Utilization**

|  | Matching including pre period acute care [main analysis] | | | Matching excluding pre period acute care [sensitivity analysis] | | |
| --- | --- | --- | --- | --- | --- | --- |
|  | estimated change in acute care | confidence interval | p-value | estimated change in acute care | confidence interval | p-value |
| ITT | -92.4 | [-113.0,-72.0] | <0.001 | -92.5 | [-113.0,-72.0] | <0.001 |
| ATET | -208.4 | [-284.0,-133.0] | <0.001 | -210.1 | [-286.0,-134.0] | <0.001 |

References

1. Medicaid.gov. *DQ atlas [Internet]. Baltimore (MD): Centers for Medicare and Medicaid Services; [cited 2023 April 1]*. Available from: <https://www.medicaid.gov/dq-atlas/>
